# Device performance enhancement via a Si-rich silicon oxynitride buffer layer for the organic photodetecting device

**DOI:** 10.1038/s41598-017-01653-z

**Published:** 2017-05-04

**Authors:** Sung Heo, Jooho lee, Seong Heon Kim, Dong-Jin Yun, Jong-Bong Park, Kihong Kim, NamJeong Kim, Yongsung Kim, Dongwook Lee, Kyu-Sik Kim, Hee Jae Kang

**Affiliations:** 1 Platform Technology Lab, Samsung Advanced Institute of Technology, 130, Samsung-ro, Yeongtong-gu, Suwon-si, Gyeonggi-do 443-803 Korea; 2 Organic Materials Laboratory, Samsung Advanced Institute of Technology, 130, Samsung-ro, Yeongtong-gu, Suwon-si, Gyeonggi-do 443-803 Korea; 3 Department of Physics, Chungbuk National University, Cheongju, 361-763 Korea

## Abstract

An advanced organic photodetector (OPD) with a butter layer of Si-rich silicon oxynitride (SiO_x_N_y_) was fabricated. The detector structure is as follows: Indium tin oxide (ITO) coated glass substrate/SiO_x_N_y_(10 nm)/naphthalene-based donor:C60(1:1)/ITO. Values of x and y in SiO_x_N_y_ were carefully controlled and the detector performances such as dark current and thermal stability were investigated. When the values of x and y are 0.16 and 0.66, the detector illustrates low dark current as well as excellent thermal stability. In the OPD, silicon oxynitride layer works as electron barrier under reverse bias, leading to the decrease of dark current and increase of detectivity. Since the band gap of silicon oxynitride unlike conventional buffer layers can also be controlled by adjusting x and y values, it can be adapted into various photodiode applications.

## Introduction

Organic photodetectors (OPDs) have been widely used in practical applications such as photo-sensors, chemical sensors^1–6^, X-ray detectors^7^, and image sensors^8–11^. Among the applications, image sensors, which have been considered of replacing conventional silicon-based image sensors^4, 10^, have attracted research interest from industries and there has been considerable effort to improve the device performances of them such as the spectral response, external quantum efficiency (EQE), dark current (DC), and sensitivity. Among these, the External quantum efficiency (EQE), DC, and thermal stability are the key factors to evaluate the performance of OPDs. EQE is coupled to the efficiency and signal-to-noise ratio (SNR) of the devices. Low DC stabilizes the signal of the devices, leading to high SNR. Because organic materials in OPDs are exposed to high temperature process such as post-annealing at 180 °C, passivation step, top layer planarization, and microlens forming process, they should be thermally stable without performance degradation.

Decreasing DC and enhancing thermal stability have been generally improved by introducing new buffer layers^12^ such as MoO_x_^13^, WO_x_^14^, and VO_x_^15^. However, MoO_x_ is susceptible to the loss of oxygen during evaporation which can result in the change of stoichiometry and electronic energy levels^16^. Since various active materials are also used in fabricating OPDs, the band structure of a buffer layer should be aligned to HOMO and LUMO levels of bulk heterojuction (BHJ) films. Conventional buffer layers such as MoO_x_ are very difficult to change their band structure, i.e. their HOMO and LUMO levels do not change without regard to neighboring active materials. Therefore, new buffer layers with thermal stability and easily adjustable band structure have been required.

As one of good candidates for new buffer layers, silicon oxynitride (SiO_x_N_y_) film has been well received because its band structure is able to be freely controlled by adjusting the x and y. Moreover, it is thermally stable at high temperature process and is also chemically inert enough to be used in electronic devices^17^ such as thin-film transistors^18^, and buffer layer for Flash memory^19^ because of their excellent passivation characteristics. Here, we report that introduction of SiO_x_N_y_ film as buffer layer into OPDs decreased DC, leading to high detectivity and photo-responsivity. In addition, the OPDs become thermally stable at high temperature process.

## Materials and Methods

**Figure 1:**
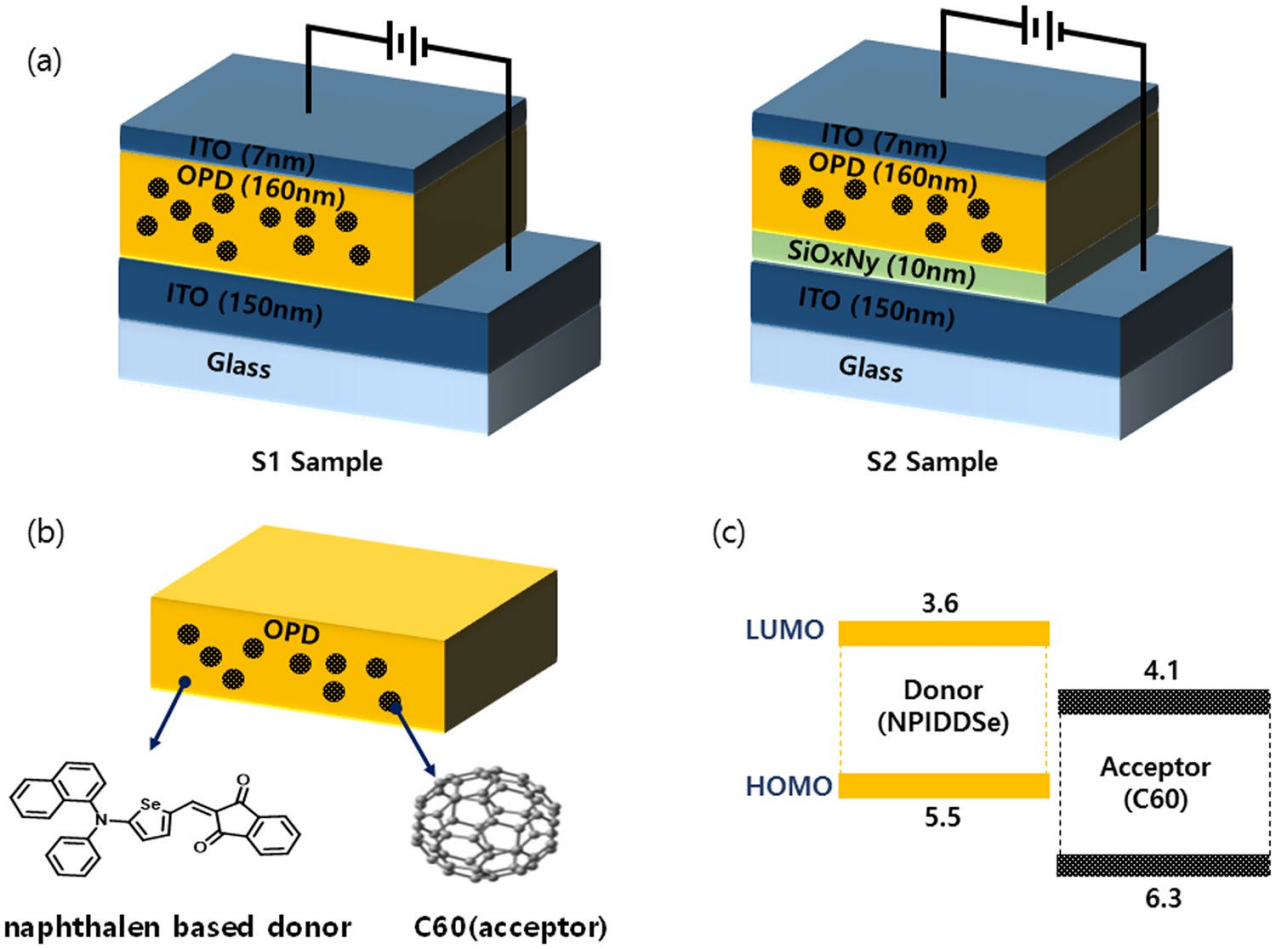
(**a**) Structure of the OPD device with and without SiOxNy buffer layer. (**b**) Chemical structure of blend organic film (**c**) HOMO and LUMO energy values of blend organic film.

Two kinds of samples were fabricated on indium tin oxide (ITO)-coated glasses. Si-rich SiO_x_N_y_ films were sequentially deposited on ITO glass by Plasma-Enhanced Chemical Vapor Deposition (PECVD) using various SiH_4_:NH_3_:NO_2_ gas mixtures with carrier N_2_ gas; 670 W of RF power was applied and the deposition temperature was 180 °C. The S1 sample does not have a SiOxNy buffer layer, while the S2 sample has 10 nm thickness of SiOxNy buffer layer, where the ratios of x (O/Si) and y (N/Si) are 0.16 and 0.66, respectively. After depositing SiOxNy buffer layer, a 160-nm-thick 1:1 organic blend layer of ‘naphthalen based donor’, C60, and a 7-nm-thick ITO capping layer were deposited, as depicted in Fig. 1(a). The ‘naphthalen based donor’ is novel push-pull-structured organic semiconducting materials, which is 5- (naphthalen-1-yl (phenyl) amino) selenophen-2-yl) methylene) -1H-indene-1,3 (2 H) -dione, with the absorption properties selective to green-light to be produced from Samsung advanced institute of technology (SAIT) as shown in Fig. 1(b) and it have a LUMO and HOMO values as a 3.6 eV and 5.5 eV as shown in Fig. 1(c) ^20^ respectively.

## Results and Discussion

**Figure 2:**
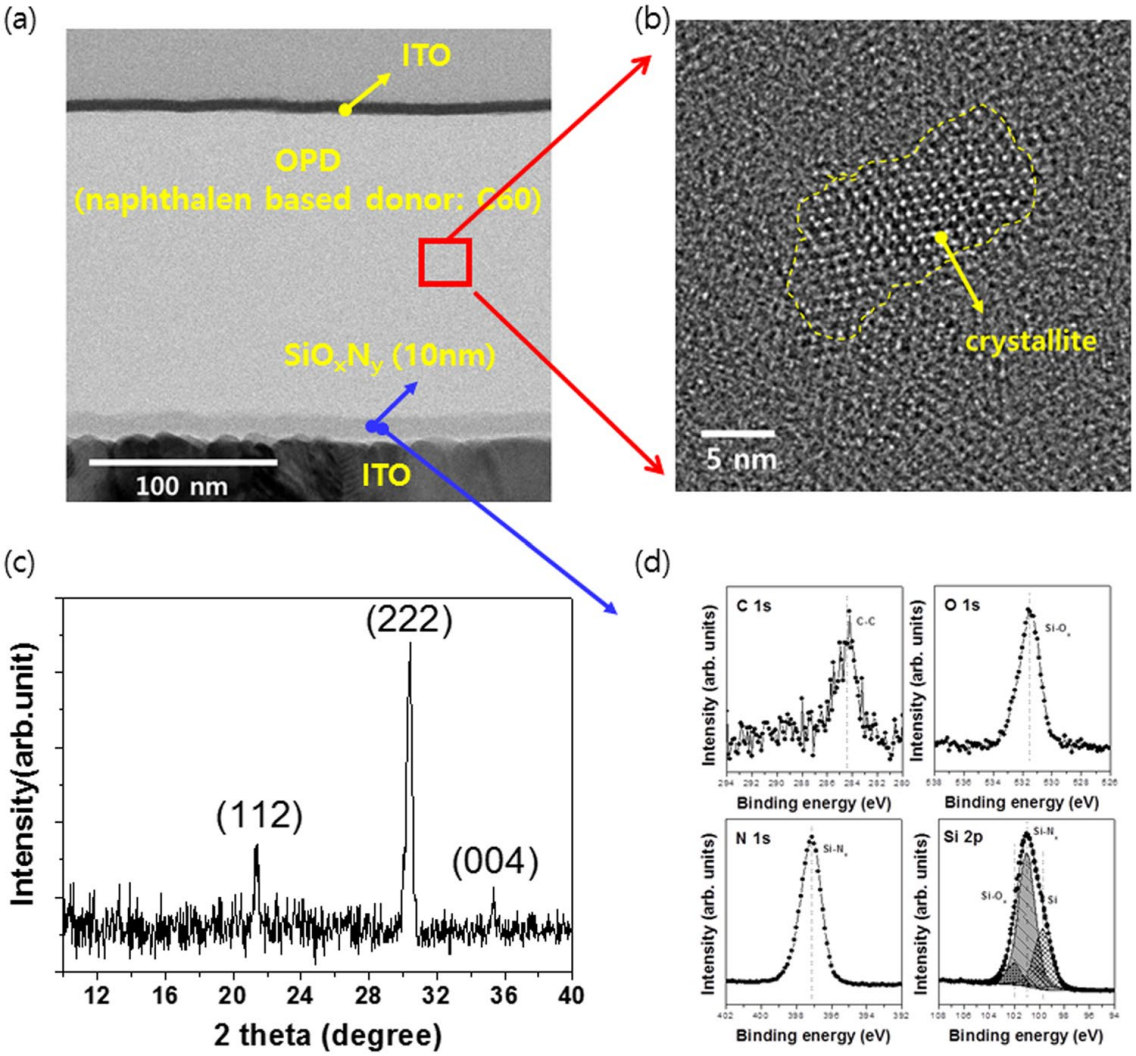
(**a**) Cross-sectional TEM image and (**b**) enlarge a rectangle of TEM image (**c**) the result of XRD (**d**) the XPS spectrum in Si-rich SiO_x_N_y_ buffer layer of the S2 sample.

After fabricating OPD samples, the thickness of SiO_x_N_y_ film in the S1 sample was characterized by Transmission Electron Microscopy (TEM). As shown Fig. 2(a), the Si-rich SiO_x_N_y_ layer on the ITO has been uniformly deposited to about 10 nm thickness. In addition, it was confirmed that Si-rich SiO_x_N_y_ layer is formed from the EDS spectrum (not shown here). The region of the active layer denoted with the red rectangle in Fig. 2(a) was enlarged in Fig. 2(b) and the layer looks partially crystalline. However, the XRD scan in Fig. 2(c) does not show any peak from the active layer but peaks from ITO. As a result, the active layer is generally amorphous phase and appears to be locally crystallized in short-range order.

Figure 2(d) shows the XPS spectrum of the Si-rich SiO_x_N_y_ film. The compositions of Si, N, and O as well as x(=O/Si) and y(=N/Si) ratios obtained from the XPS measurements are summarized in Table 1. The x and y values for the SiO_x_N_y_ sample were 0.16 and 0.66, respectively. Si 2p peak has lower peak area of Si_3_N_4_ chemical state (397.8 eV in N 1 s and 101.8 eV in Si 2p) due to the existence of Si-Si chemical state (99.6 eV in Si 2p). In general, Si 2p in SiO_2_ films is located at 103.3 eV. This shows a distinct evolution of Si 2p spectra with the incorporation of N into the dielectric film. The Si 2p peak in SiO_x_N_y_ shifts toward a low binding energy because the incorporation of N into dielectric film.

**Table 1:** Composition and electrical properties of Si-rich SiOxNy samples using AES, XPS and REELS.

Sample	XPS (after sputter 1 min)						REELS/XPS	
	Si	O	N	C	O/Si(x)	N/Si(y)	Eg(eV)	VBO for ITO
S2	54.2	8.5	35.1	2.2	0.16	0.66	3.1	0.4

**Figure 3:**
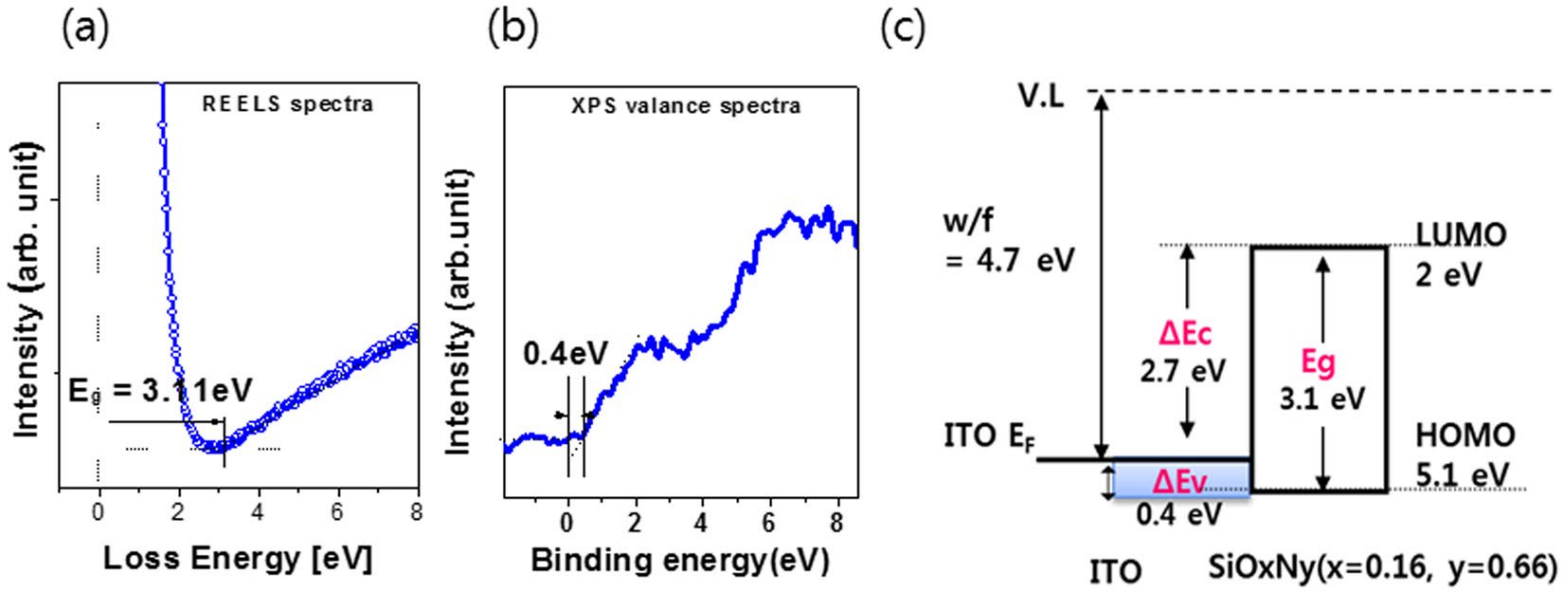
(**a**) REELS spectra (**b**) valance band spectra and (**c**) Energy band diagrams for the S2 sample.

Figure 3(a) shows REELS acquired for the Si-rich SiO_x_N_y_ thin film (Fig. 2a), and the band gap of thin film was determined from the onset value of its energy loss signal^21^. The band gaps of the SiO_x=0.16_N_y=0.66_ film was 3.1 ± 0.1 eV as shown in Fig. 3(a) although Gurava *et al*. reported^22^ that the band gap of Si-rich SiN_x_ film ranges from 2.11 eV to 2.56 eV when x varies from 0.48 to 0.87. The band gap of 3.1 eV for Si-rich SiO_x_N_y_ is much higher than that of SiN_x_ film. This discrepancy originates from the fact that the top of the valence band for a SiN_x_ film consists primarily of N 2p lone pairs while the bottom of the conduction band is dominated by the Si 3 s state^23, 24^. However, the top of the valence band of the SiO_2_ thin film is formed by a non-bonding O 2p state, and the lowest conduction band is dominated by extended Si 3 s states^25, 26^. For SiO_x_N_y_ dielectrics, the increase of O composition into the SiN_x_ modifies the valence-band density of state profile. The N 2p, O 2p nonbonding peak and Si 3 s peak lead to little change. And then, the band gap value of SiO_x_N_y_ is enlarger than that of SiN_x_.

Figure 3(b) illustrates valence band spectra acquired from the Si-rich SiO_x_N_y_ films and ITO substrate, and the valence band maximum (VBM) for each sample was determined as the intersection between the baseline and the linear fit of the leading edge of the valence band^27^. The valence band offset ΔEv was obtained as the difference between the VBMs of SiO_x_N_y_ and the Fermi energy (E_F_) of ITO substrate. There exists significant difference in the ΔEv at SiO_x_N_y_/ITO interface depending on the chemical states of SiO_x_N_y_ film. The conduction band offsets (ΔEc) were determined from the band gap (Eg) and ΔEv according to the relation ΔEc = Eg(SiO_x_N_y_) − ΔEv(SiO_x_N_y_/ITO)^28^. The energy band diagram of S2 in the SiO_x=0.16_N_y=0.66_ thin films are shown in Fig. 3(c).

**Figure 4:**
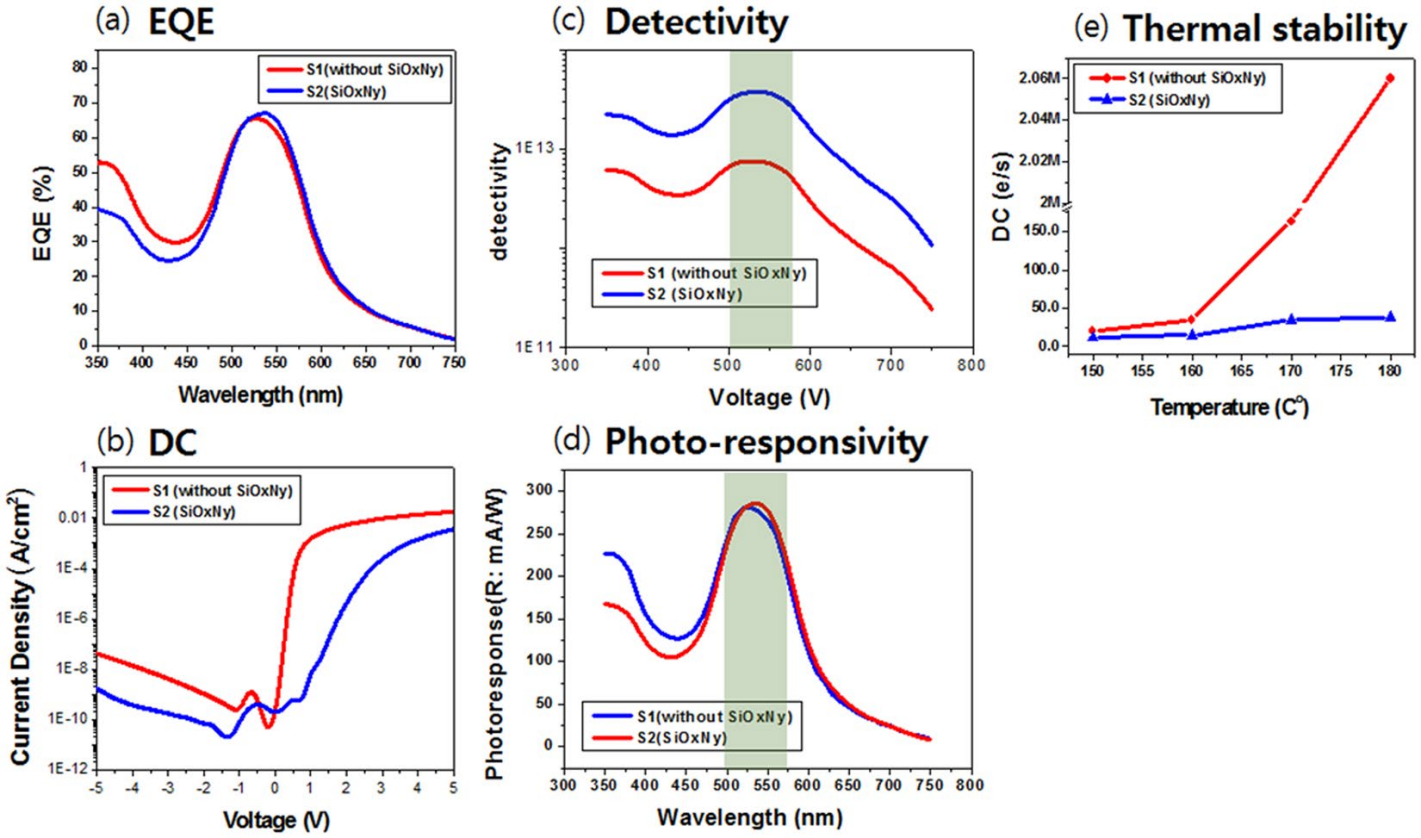
(**a**) EQE curves of S1 (without SiO_x_N_y_) and S2 (i.e with SiO_x_N_y_ film) measured for the OPDs, respectively. (**b**) Current density-voltage characteristics in the dark for the OPDs in (**a**). (**c**) Detectivities of S1 and S2. (**d**) Photo-responsivities of S1 and S2. (**e**) Thermal stability for DC with a various temperature from 150 °C to 180 °C.

Figure 4(a,b) show the results from EQE and DC for the OPD devices. In Fig. 4(a), the S2 sample exhibits equally (or slight improved) a normal EQE characteristic with a maximum value of ~67% at a wavelength of 550 nm, compared with the S1 sample (without SiO_x_N_y_ sample). The DC characteristics (0.18 nA/cm^2^ at −3 V) of the S2 sample were improved, compared with the S1 sample (4.28 nA/cm^2^ at −3 V) as shown in Fig. 4(b). For clear comparison, the units of the DC were converted to the number of electrons flowing through a unit area of 1 μm^2^ at a bias voltage of −3 V for 1 s. The calculated values are displayed near the corresponding DC curves in Fig. 4(b). The DC value for the OPD device with S2, which is 11 e/(s · μm^2^), is less than reference device (S1 sample), which is 267 e/(s · μm^2^).

According to Leem *et al*.^29^, the dark current of the OPD with MoO_x_ buffer layer was 6.41 nA/cm^2^ at −3 V. Compared with this value, the dark current of the reference sample (S1) (4.28 nA/cm^2^ at −3 V) was improved so much and the dark current of the S2 sample (0.18 nA/cm^2^ at −3 V) was enhanced enormously.

The detectivity and photo-responsivity of the OPDs are displayed in Fig. 4(c,d). The detectivity of the S1 sample (without SiO_x_N_y_) is on the order of 10^13^ in the green region, while the detectivity of the S2 sample is much higher, on the order of 10^14^ in the green region. In Fig. 4(d), the two samples show similar photo-response. The thermal stabilities of the OPDs are illustrated in Fig. 4(e). The analysis of the thermal stability of the OPD was performed by annealing the device at increasing temperatures of up to 180 °C for 30 min at each temperature. The S2 sample shows remarkably good dark current characteristic (38 e/(s · μm^2^) at −3 V, 180 °C), compared with the S1 sample (without SiO_x_N_y_ film, which has poor dark current (2,060,000 e/(s · μm^2^) at −3 V, 180 °C), as shown in Fig. 4(e). These results suggest that using SiO_x_N_y_ as a buffer layer makes OPDs thermally stable and the DC can be decreased further.

**Figure 5:**
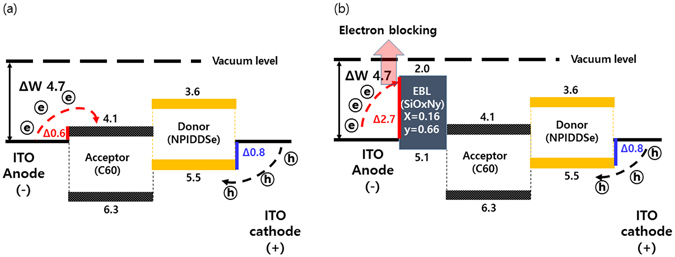
(**a**) and (**b**) Re-plots the full band diagram of without SiO_x_N_y_ (S1 sample, ITO/naphthalen based donor:C60(1:1)/ITO structure) and with SiO_x_N_y_ (S2 sample, ITO/SiO_x=0.16_N_y=0.66_/naphthalen based donor: C60(1:1)/ITO structure) using the bandgap and valance band offset of SiO_x_N_y_ films as previously measured in Fig. 3.

In order to unveil the possible origins of the reduced DC in the S2 sample, compared to reference (S1) sample, the full band diagrams of the samples are plotted in Fig. 5(a and b), based on the bandgaps and valence band offsets (Fig. 3) of the OPD without SiO_x_N_y_ (S1 sample, ITO/naphthalen based donor:C60(1:1)/ITO structure) and the sample with SiO_x_N_y_ (S2 sample, ITO/SiO_x=0.16_N_y=0.66_/naphthalen based donor:C60(1:1)/ITO structure). Both electrons and holes can transport from the ITO anode and cathode to the acceptor and donor under reverse bias condition^29, 30^ for the S1 sample (without SiO_x_N_y_ film), as shown in Fig. 5(a). The current leakage under reverse bias is primarily dominated by electrons rather than holes since the electron barrier (0.6 eV) between the work function of ITO (4.7 eV) and LUMO level (4.1 eV) of C60(1:1) in the blend system is much smaller than the hole barrier (0.8 eV) between the HOMO level (5.5 eV) of naphthalene based donor and the work function of ITO (4.7 eV).

However, the SiO_x=0.16_N_y=0.66_ film in the S2 sample acts as electron blocking role since the LUMO level of SiO_x=0.16_N_y=0.66_ has higher than that of naphthalen based donor: C60(1:1) layer. Thus, the insertion of the SiO_x=0.16_N_y=0.66_ (S2 sample) layer between the anode and acceptor layer in Fig. 5(b) increased the effective electron barrier due to its low-lying LUMO level of 2.0 eV and the barrier prevents electrons from flowing to the acceptor layer. Thus, the leakage current under reverse bias decreases.

Lastly, it is also worthwhile to mention the tunability of the bandgap of SiO_x_N_y_. According to Park, *et al*. and Wong, *et al*.^31, 32^, the energy bandgap of SiN_y_ increases from 1.1 eV to 5.5 eV as the N/Si (y) value rises in the SiN_y_ thin film. When the value of y is ~1, the valance band and the conduction band sharply decrease. However, the valance band offset (VBO) is not greatly reduced if the value is less than 1, although the conduction band offset is continuously decreased. In our previous study^33^, we reported that if y = 0.92 or less, there is a decrease in the band gap while VBO does not decrease significantly. As more oxygen atoms are added to SiN_y_, the energy bandgap of SiO_x_N_y_ increases and the valance band increases more than the conduction band. Therefore, the bandgap tunability and band structure of the SiO_x_N_y_ layer reduces the dark current and improves the device performance.

In summary, we demonstrated the device performance applied to Si-rich SiO_x_N_y_ film as a buffer layer in an OPDs. Then, external quantum efficiency (EQE) was slightly improved, and the DC and thermal stability were significantly improved, compared with that obtained without the Si-rich SiOxNy buffer layer. These results showed that a Si-rich SiOxNy could be a promising buffer layer for the practical OPD applications.

### Characterization

The surface roughness of SiO_x_N_y_ film was about 3 nm by using Atomic Force Microscopy (AFM, Dimension ICON, Bruker, not shown here). All organic layers were thermally evaporated (<10^−7^ Torr) at a rate of 1 nm/s. The overlapping area of the two electrodes was 0.04 cm^2^ (0.2 cm × 0.2 cm). The device was finally encapsulated with glass. The current-voltage characteristics of the devices were measured by a Keithley K4200 parameter analyzer. The EQE was measured using a setup illuminated by monochromatic light generated by an ozone-free xenon lamp with a chopper frequency of 30 Hz. The monochromatic light intensity was calibrated using a silicon photodiode (Hamamatsu, S1337).

Band gap and composition measurements were performed by means of reflection electron energy loss spectroscopy (REELS) using auger electron spectroscopy (AES, PHI-4700, Concentric hemispherical analyzer) and X-ray photoelectron spectroscopy (XPS, PHI Quantera II Scanning XPS Microprobe), respectively. REELS spectra were measured using the primary electron energy of 1,500 eV for excitation and constant analyzer pass energy of 10 eV. The full width at half maximum (FWHM) of the elastic peak was 0.8 eV. Before REELS analysis, samples were subjected to an Ar+ ion gun cleaning to eliminate surface contamination.
